# Effects of Dietary Probiotics and Acidifiers on the Production Performance, Colostrum Components, Serum Antioxidant Activity and Hormone Levels, and Gene Expression in Mammary Tissue of Lactating Sows

**DOI:** 10.3390/ani13091536

**Published:** 2023-05-04

**Authors:** Hongzhi Wu, Chaohua Xu, Jingjing Wang, Chengjun Hu, Fengjie Ji, Jiajun Xie, Yun Yang, Xilong Yu, Xinping Diao, Renlong Lv

**Affiliations:** 1 Tropical Crop Genetic Resource Research Institute, Chinese Academy of Tropical Agricultural Sciences, Haikou 571101, China; 2 College of Animal Science and Technology, Northeast Agricultural University, Harbin 150030, China; 3 Zhanjiang Experimental Station, Chinese Academy of Tropical Agricultural Sciences, Zhanjiang 524000, China

**Keywords:** probiotics, acidifiers, lactating sows, colostrum components, serum antioxidant activity and hormone levels, mammary tissue

## Abstract

**Simple Summary:**

Pig production heavily depends on the reproductive performance of sows. The antioxidant capacity and hormone levels of lactating sows have an essential impact on their own health and the health of future generations. We investigate the effects of dietary probiotics and acidifiers on the production performance, colostrum components, serum antioxidant activity and hormone levels, and gene expression in the mammary tissue of lactating sows. The results showed that the dietary probiotics and acidifiers improved the growth performance of piglets, decreased the serum malondialdehyde levels, increased the superoxide dismutase contents, and increased the relative expression levels of the prolactin receptor and fatty acid synthase. In conclusion, the basal diet mixed with 200 mL/d probiotics + 0.5% acidifiers improved the production performance, colostrum components, serum antioxidant activity, and hormone levels of lactating sows. This study provides a more theoretical basis for the dietary probiotics and acidifiers’ application in the diet of lactating sows and for the sustainable and healthy development of pig farming.

**Abstract:**

The aims of this study were to test the effects of dietary probiotics and acidifiers on the production performance, colostrum components, serum antioxidant activity and hormone levels, and gene expression in the mammary tissue of lactating sows. Four treatments were administered with six replicates to 24 lactating sows. The control group (GC) received a basal diet, while the experimental groups received a basal diet with 200 mL/d probiotics (GP), 0.5% acidifiers (GA), and 200 mL/d probiotics + 0.5% acidifiers (GM), respectively. Compared with the GC, (1) the average weight of the piglets on the 21st day of lactation in the GM was higher (*p* < 0.05); (2) the colostrum fat ratio increased significantly (*p* < 0.05); (3) the malondialdehyde levels in GP and GM were lower (*p* < 0.05) on the 11th day; (4) on the 1st, 11th, and 21st days, the prolactin in GP and GM increased (*p* < 0.05); (5) on the 21st day, the relative expression levels of the prolactin receptor and fatty acid synthase were increased (*p* < 0.05). In summary, the basal diet mixed with 200 mL/d probiotics + 0.5% acidifiers could improve the production performance, colostrum components, serum antioxidant activity, and hormone levels of lactating sows.

## 1. Introduction

Sow colostrum contains maternal antibodies and immunologically active cells essential for piglet survival, and new-born piglets can directly absorb and use the substances, so colostrum is vital for lactating piglets [1]. Nutrient intake during lactation largely determines piglet performance, such as birth weight and weaning litter weight [2]. Changes in the health status of the sow during gestation may affect the uniformity and consistency of the litter size [3]. The lactation ability of sows can reflect the reproductive performance of sows to a certain extent. The relationship between litter weight and sow lactation was found to have a similar pattern for different breeds of sows, i.e., litter weight at birth and litter size were positively related to sow lactation [4]. Campos et al. found that 50% of pre-weaning piglet mortality was attributed to the insufficient feed intake of lactating sows [2]. Organic acids, such as formic acid, acetic acid, propionic acid, citric acid, Yohimbe acid, lactic acid, sorbic acid, and malic acid, are widely used in animal feed additives [5].

The generally accepted organic acid action mechanism is that they lower the gastrointestinal tract pH and prolong the biochemical reaction time of nutrients in the digestive tract [6]. Probiotics are live bacterial agents in the animal intestine to maintain the intestinal microecological balance and improve its health level [7]. It contains about 20 genera of microorganisms, such as Bacillus, *Lactobacillus*, Yeast, *Bifidobacterium*, Enterococcus, Floccus, and Streptococcus [8]. It is well known that maintaining a healthy gut is critical to the digestion and absorption of nutrients in pigs. A balanced microbiota is an essential part of intestinal health. When the animal immune system is compromised, probiotics can be used as a feed additive supplement to improve intestinal health and promote nutrient digestion and absorption [9,10,11,12,13]. As natural and safe feed additives, acidifiers and probiotics can lower the intestinal pH, regulate intestinal microflora, and improve nutrient digestibility in animals [14,15]. In recent years, livestock workers have conducted more and more research on probiotics and acidifiers, especially on their application to weaned piglets. However, most acidifiers and probiotics are added separately in sow production. Little research has been reported on the development and application of their combined use on lactating sows.

Correctly applying acidifiers and probiotics is expected to be an essential method for the sustainable development of the pig industry. Therefore, the purpose of this study is to investigate the effects of adding probiotics and acidifiers and their combination on the production performance, colostrum components, serum antioxidant activity and hormones levels, and gene expression in the mammary tissue of lactating sows to provide a more theoretical basis for their application in the diet of lactating sows and to contribute to the sustainable and healthy development of pig farming.

## 2. Materials and Methods

The Chinese guidelines for animal welfare conducted this study with the animal welfare standards of the College of Animal Science and Technology, Northeast Agricultural University (NEAU-2022-0232).

### 2.1. Experiment Material

Gestating and lactating sows: Hainan Local breed sows (Tunchang), second litter, with the same genetic background and birthing date. All sows were artificially inseminated with semen from Duroc boars.

Acidifiers were supplied by Chongqing U-Power Biotechnology Co., Ltd. (Chongqing, China) mainly containing 18% phosphate, 12% ammonium formate, 15% citric acid, 15% fumaric acid, 10% lactic acid, and 30% buffer system.

Probiotics, in the liquid form, were provided by Shenzhen Baiaofei Biotechnology Co., LTD., Shenzhen, China. It was a mixture of *Lactic acid bacteria* and *Yeast*, and the effective strains and live bacteria numbers were as follows: the content of *Lactobacillus fermentum* was 1 × 10^8^ CFU/mL, and the *Basidiomycetous yeast* content was 8 × 10^7^ CFU/mL.

Enzyme-linked immunoassay kits were purchased from Shanghai Sangon Biotechnology Co., Ltd., Shanghai, China.

### 2.2. Experiment Design and Sample Collection

The experiment adopted a two-factor random design. Two levels of probiotics (0, 200 mL/d), acidifiers (0, 0.5%), and 200 mL/d probiotics + 0.5% acidifiers were selected to investigate the effects of probiotics and acidifiers on the production performance, colostrum components, serum antioxidant activity and hormones levels, and gene expression in mammary tissue of lactating sows. The basal diets for the gestating and lactating sows were a corn or soybean meal-type diet formulated according to the NRC (1998; 2012) standards for swine feeding, and the composition of the basal diets and nutritional levels were shown in Table 1. In an individual stall, 24 two-way crossbred Local breed sows were randomly divided into four groups, with six sows per replicate each. Control group (GC) received a basal diet, while the experimental groups received a basal diet with 200 mL/d probiotics (GP), 0.5% acidifiers (GA), 200 mL/d probiotics + 0.5% acidifiers (GM), respectively. The pre-trial period lasted for seven days, and the trial period lasted for 21 days. The feeding methods were wet mix with 66.67% humidity (Feed:Water = 1:2). For three days before the farrowing date, each group was fed an experimental diet restricted to 3.0 kg per day in the farrowing room, beginning on the 107th day of gestation, and fed twice a day at 8:00 and 16:00. In the first day following farrowing, every sow was fed 2.0 kg, and after that, the amount was increased by 0.5–1 kg per day. Sows were then fed almost ad libitum. Thermal insulation and warming lamps were used to keep the piglets warm. There was no restriction on drinking by sows or piglets. For cross-fostering, 11 piglets were adjusted per lactating sow, with body weight 1.45 ± 0.15 kg, in 3 days after farrowing. Then one lactating sow and 11 adjusted piglets were feeding in the individual stall. All piglets were weaned at 21 days. Aside from feeding management and immunizations, other procedures, including ventilation of the piggery, zinc (200 mg zinc lysine particles mixed in 250 kg water for daily drinking), and iron (2 mL iron dextran injection per piglet was injected into each piglet on the third day of lactation) supplementation for pigs, were carried out according to the pig farm guidelines.

Number of pigs born alive (NBPA), number of piglets live at wean (NPAW), initial litter weight of piglets (ILW), weaning litter weight of piglets (WLWP), number of pigs wean alive (NBPA), average daily feed intake (ADFI), the average weight of piglets on the first day of lactation (AVG1), the average weight of piglets on the 21st day of lactation (AVG21), an average daily gain of piglets from the first to 21st day of lactation (ADG), total lactation yield (TLY), initial backfat thickness (IBT), final backfat thickness (FBT), and BL: Backfat lose (BL) were recorded during the experiment period. Two hours after farrowing, 30 mL of colostrum was collected from the sows’ front, middle, and rear nipples, then mixed well and stored at −20 °C to determine the colostrum fat ratio (CFR), colostrum protein content (CPC), and Colostrum Lactose ratio (CLR). The CFR, CPC, and CLR in colostrum were analyzed by the FOSS milk composition rapid analyzer (MilkoScan FT120, Albania of origin). On the day of delivery birth and the 11th and 21st day of lactation, 10 mL blood was collected with promoting coagulating tubes from the ear vein of each sow. The sows ears were then disinfected with 75% alcohol and iodine tincture before blood collection, and the blood collection hole was pressed with alcohol cotton until it no longer bled after blood collection. Serum was obtained by centrifuging the blood at 3000 rpm for 15 min after resting for 15 min and stored at −20 °C for biochemical indicators testing. The concentrations of malondialdehyde (MDA), superoxide dismutase (SOD), and total antioxidant capacity (T-AOC) in serum were measured using a biochemical analyzer (Beijing Huaying Institute of Biotechnology, Beijing, China), according to the kit instructions. The concentrations of prolactin (PRL), growth hormone (GH), and insulin-like growth factor-I (IGF-1) in serum were measured by ELISA (Beijing Huaying Institute of Biotechnology), according to the kit instructions.

On the 21st day of lactation, 4 mL lidocaine hydrochloride (2 mL: 40 mg, Beijing Yimin Pharmaceutical Co., Ltd., Beijing, China) was injected into the second left papilla of the sows for local anesthesia after disinfection, and 3 g of mammary tissue samples were collected using a live sampling gun (BARD^®^ MAGNUM^®^, MG1522, Franklin, NJ, USA) and disposable sampling needles (C.R. Bard. Inc., Coington GA, USA). Following the RNA extraction method, total RNA was isolated from mammary tissue with TRIzol (Sigma, Saint Louis, MO, USA). A 2% agarose gel electrophoresis was performed to assess the quality of the RNA. Total RNA concentration and purity (A260/A280 ratio) were evaluated using an ultra-microspectrophotometer (NanoPhotometer, Implen German). A Prime Script™ RT reagent kit with gDNA Eraser (TaKaRa, Dalian, China) was used to reverse transcribe the total RNA from each mammary tissue. A TB Green™ Premix Ex Taq™ PCR kit (TaKaRa, Dalian, China) was used to amplify the cDNA. The primer sequences are shown in Table 2, and primer sequences for porcine genes were synthesized by Sangon (Shanghai, China). ABI PRISM 7500 SDS thermal cycler (Applied Biosystems, Foster City, CA, USA) was used to dispose of samples, followed by one cycle at 95 °C for 30 s, and 40 cycles of 95 °C for 5 s and 60 °C for 34 s. Based on the 2^−ΔΔCt^ method, relative gene mRNA expression was calculated and normalized by Glyceraldehyde-3-phosphate dehydrogenase and β-actin expression. All of the processes were performed in triplicates under RNase-free conditions. RT-qPCR products were cloned into pMD18-T vector (TaKaRa) and sequenced by the Sanger method. The sequencing results were compared with the gene sequences in NCBI, and the genes amplified by the primers in Table 2 were verified as the target genes according to the alignment sequence results.

### 2.3. Statistical Analysis of Data

Statistical analyses were conducted using SPSS 20.0 statistics software. All data in this study underwent the Kolmogorov–Smirnov test to check if they followed a normal distribution. Different treatments were analyzed using two-factor and multiple comparisons with Tukey’s multiple-range tests. Data were expressed as mean ± standard error of the mean and *p* < 0.05 as the significant differences.

## 3. Results

### 3.1. Effects of Probiotics and Acidifiers on the Productive Performance of Lactating Sows

The ADFI, TLY, WLWP, AVG21, and CFR in the GM were higher (*p* < 0.05) than those in GC. There were no significant differences (*p* > 0.05) among groups in AVG1, ADG, NBPA, NPAW, ILW, IBT, FBT, BL, CLR, and CPC. The primary effect analysis showed that dietary probiotics significantly increased (*p* < 0.05) the TLY, ADG and CFR, dietary acidifiers increased (*p* < 0.05) the ADFI, WLWP, and CFR, and only colostrum CFR was affected considerably (*p* < 0.05) by the dietary probiotics and acidifier interaction (Table 3).

### 3.2. Effects of Probiotics and Acidifiers on the Serum Antioxidant Activities of Lactating Sows

On the 1st day of lactation, the T-AOC contents in GM were significantly higher (*p* < 0.05) than that in GC. The MDA contents in the treatment groups were significantly lower (*p* < 0.05) than those in GC. Compared with those in GC, the SOD contents in GM and GP were higher (*p* < 0.05). The primary effect analysis showed that dietary probiotics significantly increased (*p* < 0.05) the serum T-AOC and SOD contents and decreased (*p* < 0.05) the serum MDA content of lactation sows, and dietary acidifiers significantly reduced (*p* < 0.05) the serum MDA contents of lactation sows. The dietary probiotics and acidifier interaction did not substantially affect (*p* > 0.05) the serum MDA, SOD, and T-AOC contents (Table 4).

On the 11th day of lactation, there were no significant differences (*p* > 0.05) in the T-AOC contents among the groups. Compared to those in GC, the MAD contents in the GP and GM were lower (*p* < 0.05), and the SOD contents in the GP and GM were higher (*p* < 0.05). The primary effect analysis showed that the dietary probiotics significantly decreased (*p* < 0.05) the serum MDA content and increased (*p* < 0.05) the serum SOD content of lactation sows, and the dietary acidifier significantly increased (*p* < 0.05) the serum T-AOC content of lactation sows. The serum MDA, SOD, and T-AOC contents were not significantly affected (*p* > 0.05) by the dietary probiotics and acidifier interaction (Table 4).

On the 21st day of lactation, the MAD contents of GA and GM were lower (*p* < 0.05), compared with those in GC, and the SOD contents in the GP and GM were higher (*p* < 0.05). The primary effect analysis showed that the dietary probiotics and acidifier significantly decreased (*p* < 0.05) the serum MDA content and increased (*p* < 0.05) the serum SOD content of lactation sows. The serum SOD content was significantly affected (*p* < 0.05) by the dietary probiotics and acidifier interaction (Table 4).

### 3.3. Effects of Probiotics and Acidifiers on Hormones in the Serum of Lactating Sows

On the 1st day of lactation, the IGF-1 and GH contents among groups did not differ significantly (*p* > 0.05). A higher (*p* < 0.05) PRL content was found in the GP, GA, and GM than in the GC. The primary effect analysis showed that the dietary probiotics and acidifiers significantly increased (*p* < 0.05) the serum PRL content of lactation sows. The serum IGF-1, GH, and PRL contents were not significantly affected (*p* > 0.05) by the dietary probiotics and acidifier interaction (Table 5).

On the 11th day of lactation, the GH contents among the groups did not differ significantly (*p* > 0.05). Compared with those in GC, the PRL contents in the GP and GM were higher (*p* < 0.05), and the IGF-1 contents in the GP, GA, and GM were higher (*p* < 0.05). The primary effect analysis showed that dietary probiotics significantly increased (*p* < 0.05) the serum PRL content of lactation sows, and the dietary acidifiers increased (*p* < 0.05) the serum PRL and IGF-1 contents of lactation sows. The serum PRL, IGF-1, and GH contents were not significantly affected (*p* > 0.05) by the dietary probiotics and acidifier interaction (Table 5).

On the 21st day of lactation, the GH contents among groups did not differ significantly (*p* > 0.05). Compared with those in GC, the PRL contents in the GA and GM were higher (*p* < 0.05), and the IGF-1 contents in the GA, GP, and GM were higher (*p* < 0.05). The primary effect analysis showed that dietary probiotics significantly increased (*p* < 0.05) the serum PRL and IGF-1 contents of lactation sows, and the dietary acidifiers increased (*p* < 0.05) the serum PRL and GH contents of lactation sows. The dietary probiotics and acidifier interaction did not affect (*p* > 0.05) the serum PRL, IGF-1, and GH contents (Table 5).

### 3.4. Effects of Probiotics and Acidifiers on Mammary Tissue Gene Expression of Lactating Sows

**Figure 1:**
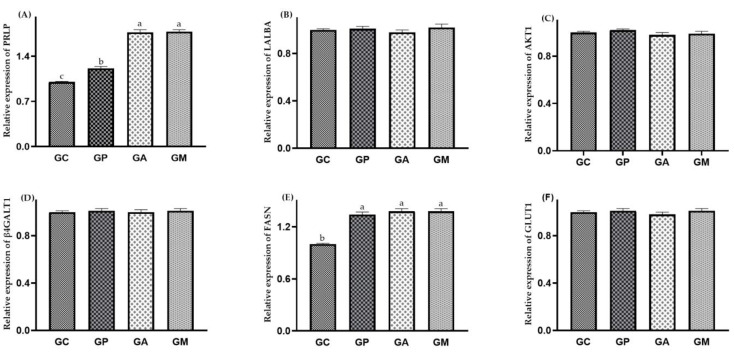
The relative expression levels of genes in mammary tissue of lactating sows. (**A**) The data of relative expression levels of *PRLP* in GC, GP, GA, and GM were 1.00 ± 0.01 ^c^, 1.21 ± 0.03 ^b^, 1.77 ± 0.04 ^a^, 1.78 ± 0.03 ^a^, individually; (**B**) the data of relative expression levels of *LALBA* from in GC, GP, GA, and GM were 1.00 ± 0.01, 1.01 ± 0.02, 0.98 ± 0.02, 1.02 ± 0.03, individually; (**C**) the data of relative expression levels of *AKT1* in GC, GP, GA, and GM were 1.00 ± 0.01, 1.02 ± 0.01, 0.98 ± 0.02, 0.99 ± 0.02, individually; (**D**) the data of relative expression levels of *β4GALT1* in GC, GP, GA and GM were 1.00 ± 0.01, 1.01 ± 0.02, 1.00 ± 0.02, 1.01 ± 0.02, individually; (**E**) the data of relative expression levels of *FASN* in GC, GP, GA, and GM were 1.00 ± 0.01 ^b^, 1.34 ± 0.03 ^a^, 1.38 ± 0.03 ^a^, 1.38 ± 0.03 ^a^, individually; (**F**) the data of relative expression levels of *GLUT1* in GC, GP, GA and GM were 1.00 ± 0.01, 1.01 ± 0.02, 0.98 ± 0.02, 1.01 ± 0.02, individually.

On the 21st day of lactation, it can be seen that the relative expression levels of *PRLP* and *FASN* were increased (*p* < 0.05) in the treatment groups compared with the GC. There were no significant differences (*p* > 0.05) in the relative expression levels of *LALBA*, *AKT1*, *β4GALT1*, and *GLUT1* among the groups (Figure 1).

## 4. Discussion

### 4.1. Effects of Probiotics and Acidifiers on the Production Performance of Lactating Sows

The lactation performance of sows reflects not only the reproductive performance of sows, but also the growth and development of piglets and the production efficiency of pig farms [1]. It was found that acidifiers significantly increased the ADFI of sows during lactation compared to the control group [16], increasing weight gain and improving the feed conversion rate of weaned piglets [17,18]. Probiotics significantly improved sow performance, increasing sow feed intake during lactation and increasing the live piglet number, the new-born piglet birth weight [19], and the average body weight of weaned piglets [20]. This study showed that dietary probiotics significantly improved the feed intake of sows and the average weight of weaned piglets [21]. Feeding probiotics, with *Bacillus licheniformis* and *Bacillus subtilis*, to lactating sows significantly increased their litter size and weaned piglet weight while reducing diarrhea rates and pre-weaning mortality [22,23]. The supplementation of acidifiers and probiotics in this study significantly increased the ADFI of sows, TLY, AVG21, and WLWP, consistent with previous studies. This may be related to the ability of the acidifiers to mask undesirable odors in feeds, improve the palatability of feeds, and thus increase the feed intake of pigs [24,25]. In addition, acidifiers inhibit the growth of harmful bacteria by lowering the sow intestine’s pH and help probiotics to create a dominant flora. The dietary probiotics fed in this study contained acid-tolerant bacteria such as *Lactic acid bacteria*. The proper acidity helps their proliferation in the gut of sows, and their acid production also helps stabilize and increase the acidifying effect of the acidifier in the digestive tract [26]. However, it has also been shown that adding citric acid alone has no significant impact on the feed intake and body weight gain of sows [27]. Acidifiers were widely used in livestock production, but the results of the studies on the effects of acidifiers on sow feed intake were not consistent. This may be due to the animal breed and age, feeding method, type and dosage of acidifiers and probiotics, feed type, dietary acid binding ability, and/or other factors [28].

### 4.2. Effects of Probiotics and Acidifiers on the Colostrum Composition of Lactating Sows

Milk from lactating sows is the direct energy source and protein for suckling piglets [29]. A change in milk composition can affect health status and immunity and directly affect the suckling piglet’s growth and development [30]. Liu et al. [27] found that citric acid significantly increased the milk’s protein contents, IgM and IgA, in the colostrum of lactating sows. Overland et al. [31] found that 0.8% and 1.2% potassium diformate, an acidifier, had the function of increasing the milk’s fat content during the lactation of sows. Chen et al. [32] found that dietary potassium butyrate significantly increased the fat, protein, and lactose contents in the milk of lactating sows, indicating that potassium butyrate could improve milk quality. The dietary supplementation of 500 mg/kg coated sodium butyrate increased milk fat and total milk solids by 29.75% and 10.94%, respectively [33]. Agazzi et al. [34] concluded that some probiotics, such as *Bacillus*, *Lactobacillus,* and *Streptococcus*, improved colostrum quality, milk quality, litter size and viability, and piglet weight. The results of this study were similar to the previous part. The contents of CFR in the colostrum of all treated groups were significantly increased, which was consistent with the results of the relative expression levels of *FASN* among all treated groups, indicating that probiotics and acidifiers could partially improve milk quality by increasing the CFR in the colostrum. The CLR data in the colostrum was consistent with the results of the relative expression levels of *β4GALT1* and *GLUT1*, and the CPC data in the colostrum was consistent with the results of the relative expression levels of *AKT1* and *LALBA*.

### 4.3. Effects of Probiotics and Acidifiers on the Serum Antioxidant Activity of Lactating Sows

The antioxidant capacity is closely related to the animal health degree [35]. MDA, T-AOC, and SOD activity can reflect the antioxidant activity of animals, and MDA can be used as an essential indicator to detect the degree of oxidative damage in the animal body. Ma et al. [36] found that organic acid, which primarily contains formic acid, formate ammonia and propionate, significantly reduced the serum MDA levels and significantly increased the serum SOD contents of animals, indicating that the acidifier could promote the improvement of the oxidation resistance of animals. Xu et al. [37] found that acidifiers, which primarily contain formic acid and acetic acid, regulated the serum T-AOC and SOD activity of weaned piglets. Zhang et al. [38] found that the probiotics, which primarily contain *Lactobacillus casei*, *Lactobacillus acidophilus*, *Bifidobacterium bifidum*, and *Enterococcus faecium*, significantly reduced the MDA concentration in male and female broilers at 21 days of age and significantly increased the total SOD levels in the blood, indicating that probiotics improved the blood antioxidant capacity of animals. Wang et al. [39] found that compound probiotics, which primarily contain microencapsulated *Enterococcus faecium*, microencapsulated *Lactobacillus plantarum*, and *Bacillius subt*, significantly improved antioxidant activity and substantially increased the serum T-AOC levels of broilers. The results of this study were the same as these of the above research. In this study, the MDA content in the sow serum was significantly reduced, and it greatly increased the serum SOD activity in the GM of lactating sows, indicating that the combination of probiotics and acidifiers can improve the antioxidant function, which is beneficial to the growth of animals. It may be that the probiotics have the effect of activating SOD [40], or it may be that the probiotics themselves could produce SOD in the metabolic process, prompting the antioxidant production [41], reducing the free radical damage, and forming a solid dominant flora in the intestine, which helps the digestion and absorption of nutrients in the intestine.

### 4.4. Effects of Probiotics and Acidifiers on the Serum Hormone Levels of Lactating Sows

The lactation process includes initiating and maintaining lactation, regulated by various hormones such as estrogen, progesterone, and prolactin [42]. Therefore, lactation yield is an important index to measure the production performance of sows. However, there are few studies on the effects of probiotics and acidifiers on the PRL content of lactating sows. In this study, compared with the control group, the PRL contents in the serum of lactating sows were increased in GP, GA, and GM; the reason may be that the probiotics and acidifiers stimulate the expression of *PRLR* in the mammary tissue, which increased the PRL content. This was consistent with the relative expression levels of *PRLR* in the mammary tissue of lactating sows.

IGF-I is essential in mammary gland development and lactation [43]. It can promote gastrointestinal tissue growth in newborn animals, accelerate the maturation of gastrointestinal function, and plays an essential role in regulating glucose and lipid metabolism and promoting cell proliferation and differentiation. In addition, IGF-I can improve the utilization of amino acids in protein synthesis [44]. It proved that sorbic acid could significantly increase the serum IGF-I concentration of sows and piglets on the 7th day of lactation and promote the growth and development of weaned piglets [45]. Murugesan et al. [46] found that lactic acid bacteria increased the serum IGF-1 level of nursery pigs. In this study, as in previous studies, it was found that probiotics, acidifiers, and mixtures of both increased the serum IGF-I levels on day 21 of lactating sows. Furthermore, the IGF-I levels in lactating sows tended to increase with the number of lactation days, similar to the average daily weight gain and litter weight in this study.

GH is one of the most important hormones to promote animal growth [47]. It is synthesized and secreted by the anterior pituitary gland of animals, which can promote protein synthesis and inhibit protein degradation. Lincoln et al. [48] found that the growth hormone receptor expression was very high in the mouse mammary gland cell proliferation and lactation stage. This indicates that GH directly acted on the animal mammary glands to promote cell proliferation and lactation. Du et al. [49] studied the effect of probiotics, primarily containing *Bacillus amyloliquefaciens C-1*, on the serum biochemical hormone levels of the calf and found that the impact of adding probiotics on the serum GH levels of cows was not significantly different. However, there was a tendency for the treated groups in this study to increase the GH levels in sow serum, which is consistent with the milk production of each treated group. Therefore, it is inferred that the mixtures of probiotics and acidifiers promoted lactation in sows by increasing serum lactogen levels.

## 5. Conclusions

In this study, the optimum dosage is a 200 mL/d probiotics and 0.5% acidifiers mixture added to the diets of lactating sows, which can improve the production performance, colostrum components, serum antioxidant activity and hormone levels, and gene expression in the mammary tissue of lactating sows.

## Figures and Tables

**Table 1:** Composition (kg/100 kg) of the basal experimental diets ^1^ for gestating and lactating sows.

Items	Content
Ingredients	
Corn	69.00
Wheat bran	3.30
Soybean meal	19.00
Fish meal	2.00
Soybean oil	2.60
Calcium hydrogen phosphate	0.40
Limestone	1.00
Salt	0.70
Premix ^2^	2.00
Total, kg	100.00
Nutrient levels, on an air-dry basis:	
Digestible energy ^3^, DE, MJ/kg	13.98
Crude protein ^4^, CP, %	16.35
Calcium ^4^, Ca, %	0.73
Phosphorus ^4^, P, %	0.34
Lysine ^4^, Lys, %	0.92
Methionine ^4^, Met, %	0.26
Threonine ^4^, Thr, %	0.59

^1^ Based on the NRC (1998; 2012) nutrient requirements for lactating sows. ^2^ The premix provided the following per kg of diet: VA 2000 IU, VD 200 IU, VE 45 IU, VK 0.5 mg, VB_1_ 1 mg, pantothenic acid 12 mg, nicotinic acid 10.25 mg, VB_6_ 3.85 mg, VB_12_ 15 ug, folic acid 1.35 mg, biotin 0.21 mg, VC 200 mg, Mn as manganese sulfate 20 mg, Fe as ferrous sulfate 80 mg, Cu as copper sulfate 5 mg, I as potassium iodide 0.14 mg, Se as sodium selenite 0.15 mg. ^3^ Calculated value (NRC, 1998; 2012). ^4^ Analyzed content.

**Table 2:** Primer Sequence list.

Gene	Gene Name	Forward and Reverse Primers	Product Size	Accession No.
PRLP	Prolactin receptor	F:5′-GGCTCCGTTTGAAGAACCAA-3′	67	NM_001001868.1
		R:5′-GTCTTTCGCAGCTGGATTCTG-3′		
LALBA	Alpha-Lactalbumin	F: 5′-GTGGTGGGGATTCTCTTTCC-3′	179	NM_214360
		R: 5′-TCTGTGCTGCCATTGTCATG-3′		
AKT1	Serine/Threonine kinases	F: 5′-CCTGAAGAAGGAGGTCATCG-3′	81	NM_001159776
		R: 5′-TCGTGGGTCTGGAAGGAGTA -3′		
β4GALT1	Bata-1,4-galactosyltransferase 1	F: 5′-GAGTTTAACATGGCGTGGAC-3′	185	XM003130680
		R: 5′-TGACGCTGTAGGATTGGGTG-3′		
FASN	Fatty acid synthase	F: 5′-GCTTGTCCTGGGAAGAGTGTA-3′	115	NM001099930
		R: 5′-AGGAACTCGGACATAGCGG-3′		
GLUT1	Glucose transporter	F: 5′-GATGAAGGAGGAGTGCCG-3′	106	EU012358
		R: 5′-CAGCACCACGGCGATGAGGAT-3′		
GAPDH	Glyceraldehyde-3-phosphate dehydrogenase	F: 5′-GTCGGAGTGAACGGATTTGG-3′	76	NM_001206359.1
		R: 5′-CAATGTCCACTTTGCCAGAGTTAA-3′		
β-actin	-	F: 5′-AGGCTACAGCTTCACCACCAC-3′	95	AB618546
		R: 5′-CCATCTCCTGCTCAAAATCCA-3′		

**Table 3:** Effects of probiotics and acidifiers on the production performance and colostrum composition of lactating sows.

Items	Groups				*p*-Value	Main Effect Analysis						
	GC	GP	GA	GM		Probiotic, mL/d		Acidifier, g/kg		*p*-Value		
						0	200	0	0.5	GP	GA	GP × GA
ADFI, kg	6.49 ± 0.37 ^b^	6.64 ± 0.55 ^ab^	6.70 ± 0.65 ^ab^	7.17 ± 0.49 ^a^	0.047	6.31 ± 0.35	6.62 ± 0.31	6.38 ± 0.28 ^c^	6.81 ± 0.30 ^d^	0.081	0.038	0.434
TLY, kg	179 ± 16.04 ^b^	206 ± 39.10 ^ab^	201 ± 26.87 ^ab^	227 ± 28.26 ^a^	0.045	187 ± 15.73 ^c^	214 ± 17.85 ^d^	189 ± 18.38	211 ± 27.07	0.017	0.074	0.925
AVG1, kg	1.58 ± 0.35	1.32 ± 0.21	1.53 ± 0.31	1.78 ± 0.55	0.109	1.36 ± 0.12	1.35 ± 0.29	1.25 ± 0.13	1.46 ± 0.26	0.083	0.098	0.073
AVG21, kg	6.73 ± 0.21 ^b^	6.74 ± 0.35 ^b^	6.76 ± 0.52 ^b^	7.48 ± 0.67 ^a^	0.045	6.38 ± 0.53	6.74 ± 0.86	6.37 ± 0.43	6.75 ± 0.89	0.085	0.073	0.079
ADG, g	225 ± 14.21	238 ± 12.38	229 ± 22.97	254 ± 32.13	0.315	227 ± 18.52 ^b^	248 ± 20.55 ^a^	231 ± 14.84	240 ± 29.31	0.048	0.382	0.482`
NBPA	10.96 ± 0.88	11.13 ± 1.43	11.46 ± 1.64	11.67 ± 1.37	0.740	11.21 ± 1.29	11.41 ± 1.41	11.10 ± 1.10	11.68 ± 1.46	0.714	0.435	0.821
NPAW	9.21 ± 0.95	10.44 ± 1.97	10.61 ± 0.95	10.94 ± 0.86	0.357	10.21 ± 1.16	10.79 ± 1.49	10.13 ± 1.65	10.88 ± 0.99	0.259	0.153	0.815
ILW, kg	15.98 ± 2.80	14.31 ± 2.82	15.90 ± 3.14	17.19 ± 2.19	0.429	15.86 ± 2.87	15.76 ± 2.87	15.07 ± 2.82	16.56 ± 2.69	0.975	0.243	0.301
WLWP, kg	59.03 ± 5.94 ^b^	64.21 ± 12.37 ^ab^	65.58 ± 8.73 ^ab^	75.10 ± 7.92 ^a^	0.041	62.49 ± 8.07	69.83 ± 11.69	61.70 ± 9.82 ^c^	70.52 ± 9.62 ^d^	0.058	0.031	0.612
IBT, mm	21.57 ± 2.90	22.24 ± 1.65	22.41 ± 1.41	24.07 ± 0.99	0.097	21.99 ± 2.25	23.16 ± 1.68	21.91 ± 2.30	23.24 ± 1.52	0.151	0.071	0.471
FBT, mm	18.21 ± 2.09	18.82 ± 1.52	18.65 ± 0.99	20.48 ± 1.66	0.083	18.48 ± 1.59	19.65 ± 1.67	18.57 ± 1.77	19.57 ± 1.66	0.075	0.120	0.319
BL, mm	3.36 ± 0.41	3.19 ± 0.38	3.03 ± 0.61	2.86 ± 0.75	0.529	3.48 ± 0.80	3.32 ± 0.87	3.57 ± 0.56	3.23 ± 0.84	0.652	0.361	0.791
CFR, %	2.08 ± 0.12 ^b^	2.98 ± 0.75 ^a^	2.88 ± 0.53 ^a^	3.38 ± 0.18 ^a^	0.002	2.54 ± 0.62 ^c^	3.24 ± 0.63 ^d^	2.49 ± 0.68 ^e^	2.99 ± 0.33 ^f^	0.003	0.004	0.032
CLR, %	7.79 ± 0.32	9.08 ± 2.67	9.30 ± 0.75	9.63 ± 0.24	0.172	8.76 ± 1.12	9.38 ± 1.31	8.48 ± 1.95	9.39 ± 0.72	0.178	0.087	0.575
CPC, %	9.33 ± 2.67	9.91 ± 1.41	9.66 ± 1.36	10.11 ± 1.37	0.889	9.39 ± 1.94	9.89 ± 1.15	9.52 ± 1.99	9.77 ± 1.09	0.448	0.709	0.926

Note: ADFI: Average Daily Feed Intake; TLY: Total lactation yield; AVG1: Average weight of piglets on the first day of lactation; AVG21: Average weight of piglets on the 21st day of lactation; ADG: the average daily gains of piglets from the first to 21st day of lactation; NBPA: Number of pigs born alive; NPAW: Number of piglets live at wean; ILW: Initial litter weight; WLWP: Weaning litter weight of piglets; IBT: Initial backfat thickness; FBT: Final backfat thickness; BL: Backfat lose. CFR: Colostrum fat ratio; CLR: Colostrum Lactose ratio; CPC: Colostrum protein content. ^a–f^ In the same row, values with the same small or no letter superscripts mean no significant difference (*p* > 0.05), and with a different small letter, superscripts mean significant difference (*p* < 0.05).

**Table 4:** Effects of probiotics and acidifiers on antioxidant activity in the serum of lactation sows.

Items	Groups				*p*-Value	Main Effect Analysis						
	GC	GP	GA	GM		Probiotic, mL/d		Acidifier, g/kg		*p*-Value		
						0	200	0	0.5	GP	GA	GP × GA
1st												
T-AOC, U/mL	10.25 ± 0.20 ^b^	10.39 ± 0.23 ^b^	10.34 ± 0.27 ^b^	10.89 ± 0.21 ^a^	0.035	10.29 ± 0.23 ^c^	10.64 ± 0.43 ^d^	10.32 ± 0.22	10.61 ± 0.55	0.038	0.071	0.191
MDA, nmol/L	4.84 ± 0.15 ^a^	4.59 ± 0.15 ^b^	4.52 ± 0.11 ^b^	4.29 ± 0.10 ^c^	<0.001	4.68 ± 0.21 ^f^	4.44 ± 0.20 ^g^	4.72 ± 0.20 ^d^	4.41 ± 0.15 ^e^	<0.001	<0.001	0.825
SOD, U/mL	69.90 ± 0.49 ^c^	71.54 ± 0.45 ^b^	69.94 ± 0.45 ^c^	72.41 ± 0.53 ^a^	<0.001	69.92 ± 0.62 ^e^	71.98 ± 0.70 ^d^	70.72 ± 1.09	71.17 ± 1.37	<0.001	0.083	0.112
11th												
T-AOC, U/mL	16.73 ± 0.55	17.02 ± 0.36	17.20 ± 0.47	17.38 ± 0.39	0.107	16.96 ± 0.54	17.20 ± 0.40	16.87 ± 0.47 ^e^	17.29 ± 0.42 ^f^	0.211	0.034	0.760
MDA, nmol/L	4.64 ± 0.38 ^a^	3.60 ± 0.12 ^b^	4.43 ± 0.67 ^a^	3.29 ± 0.05 ^b^	<0.001	4.54 ± 0.53 ^c^	3.44 ± 0.18 ^d^	4.12 ± 0.61	3.86 ± 0.75	<0.001	0.121	0.782
SOD, U/mL	73.26 ± 0.80 ^c^	74.31 ± 0.20 ^b^	73.41 ± 0.56 ^c^	75.54 ± 0.90 ^a^	<0.001	73.34 ± 0.66 ^d^	75.05 ± 0.81 ^e^	73.91 ± 0.88	74.48 ± 1.32	<0.001	0.053	0.148
21st												
T-AOC, U/mL	22.34 ± 0.36	22.38 ± 0.65	22.42 ± 0.44	22.59 ± 0.86	0.897	22.3 ± 0.39	22.48 ± 0.74	22.36 ± 0.50	22.50 ± 0.66	0.675	0.568	0.787
MDA, nmol/L	4.08 ± 0.41 ^a^	3.89 ± 0.03 ^ab^	3.62 ± 0.09 ^bc^	3.54 ± 0.04 ^c^	0.002	3.81 ± 0.40 ^d^	3.99 ± 0.11 ^e^	3.90 ± 0.20 ^f^	3.63 ± 0.12 ^g^	0.041	0.001	0.961
SOD, U/mL	72.32 ± 0.77 ^c^	74.68 ± 0.75 ^b^	72.3 ± 0.44 ^c^	78.28 ± 0.88 ^a^	<0.001	72.34 ± 0.60 ^d^	76.48 ± 2.04 ^e^	73.50 ± 1.43 ^f^	75.32 ± 2.16 ^g^	<0.001	<0.001	<0.001

Note: T-AOC: total antioxidant capacity; MDA: Malondialdehyde; SOD: Superoxide dismutase. ^a–g^ In the same row, values with the same small or no letter superscripts mean no significant difference (*p* > 0.05), and with a different small letter, superscripts mean significant difference (*p* < 0.05).

**Table 5:** Effects of probiotics and acidifiers on serum hormone levels of lactating sows.

Items	Groups				*p*-Value	Main Effect Analysis						
	GC	GP	GA	GM		Probiotic, mL/d		Acidifier, g/kg		*p*-Value		
						0	200	0	0.5	GP	GA	GP × GA
1st												
PRL, μIU/mL	214 ± 1.59 ^c^	217 ± 1.26 ^b^	224 ± 2.05 ^a^	224 ± 1.89 ^a^	<0.001	214 ± 5.64 ^g^	220 ± 4.29 ^f^	215 ± 2.10 ^e^	224 ± 1.89 ^d^	0.020	<0.001	0.080
IGF-1, ng/mL	181 ± 3.02	183 ± 3.45	184 ± 3.72	185 ± 3.95	0.313	182 ± 3.52	184 ± 3.71	182 ± 3.22	184 ± 3.71	0.333	0.111	0.857
GH, ng/mL	5.40 ± 0.03	5.49 ± 0.23	5.66 ± 0.70	5.81 ± 0.72	0.540	5.53 ± 0.49	5.65 ± 0.53	5.45 ± 0.16	5.74 ± 0.68	0.575	0.185	0.893
11th												
PRL, μIU/mL	236 ± 3.14 ^c^	246 ± 3.30 ^b^	238 ± 3.87 ^c^	258 ± 2.36 ^a^	<0.001	237 ± 3.53 ^d^	252 ± 6.81 ^e^	241 ± 5.81 ^f^	248 ± 5.53 ^g^	<0.001	<0.001	0.001
IGF-1, ng/mL	198 ± 1.66 ^b^	201 ± 1.26 ^a^	202 ± 1.74 ^a^	201 ± 1.31 ^a^	0.001	200 ± 2.59	201 ± 1.23	199 ± 2.16 ^d^	201 ± 1.52 ^c^	0.065	0.005	0.005
GH, ng/mL	6.21 ± 0.52	6.38 ± 0.23	6.36 ± 0.20	6.53 ± 0.16	0.391	6.28 ± 0.38	6.45 ± 0.21	6.29 ± 0.39	6.44 ± 0.20	0.202	0.247	0.997
21st												
PRL, μIU/mL	226 ± 3.40 ^c^	236 ± 4.92 ^b^	230 ± 7.27 ^b^	249 ± 7.38 ^a^	<0.001	228 ± 5.79 ^e^	243 ± 9.22 ^d^	231 ± 6.42 ^f^	240 ± 12.16 ^g^	<0.001	0.002	0.066
IGF-1, ng/mL	187 ± 0.49 ^c^	195 ± 2.84 ^a^	190 ± 1.93 ^b^	194 ± 1.43 ^a^	<0.001	188 ± 1.80 ^e^	195 ± 2.17 ^d^	192 ± 4.49	192 ± 2.99	<0.001	0.296	0.070
GH, ng/mL	5.69 ± 0.58	6.06 ± 0.61	6.12 ± 0.64	6.65 ± 0.50	0.067	5.90 ± 0.62	6.36 ± 0.61	5.87 ± 0.60 ^a^	6.58 ± 0.61 ^b^	0.071	0.044	0.747

Note: PRL: prolactin; IGF-1: insulin-like growth factors -1; GH: growth hormone. ^a–g^ In the same row, values with the same small or no letter superscripts mean no significant difference (*p* > 0.05), and with a different small letter, superscripts mean significant difference (*p* < 0.05).

## Data Availability

Data presented are original and not inappropriately selected, manipulated, enhanced, or fabricated.
